# Metataxonomic analysis of halophilic archaea community in two geothermal oases in the southern Tunisian Sahara

**DOI:** 10.1093/femsle/fnae106

**Published:** 2024-12-05

**Authors:** Afef Najjari, Khaled Elmnasri, Hanene Cherif, Stephen Burleigh, Amel Guesmi, Mouna Mahjoubi, Javier A Linares-Pastén, Ameur Cherif, Hadda-Imene Ouzari

**Affiliations:** Faculté des Sciences de Tunis, LR03ES03 Laboratoire de Microbiologie et Biomolécules Actives, Université Tunis El Manar, 2092 Tunis, Tunisia; Higher Institute for Biotechnology, University Manouba, BVBGR-LR11ES31, Biotechpole Sidi Thabet, 2020 Ariana, Tunisia; Higher Institute for Biotechnology, University Manouba, BVBGR-LR11ES31, Biotechpole Sidi Thabet, 2020 Ariana, Tunisia; Department of Process and Life Science Engineering, Lund University, PO Box 124, 22100 Lund, Sweden; Higher Institute for Biotechnology, University Manouba, BVBGR-LR11ES31, Biotechpole Sidi Thabet, 2020 Ariana, Tunisia; Higher Institute for Biotechnology, University Manouba, BVBGR-LR11ES31, Biotechpole Sidi Thabet, 2020 Ariana, Tunisia; Division of Biotechnology, Faculty of Engineering, Lunds Tekniska Högskola (LTH), Lund University, P. O. Box 124, 22100 Lund, Sweden; Higher Institute for Biotechnology, University Manouba, BVBGR-LR11ES31, Biotechpole Sidi Thabet, 2020 Ariana, Tunisia; Faculté des Sciences de Tunis, LR03ES03 Laboratoire de Microbiologie et Biomolécules Actives, Université Tunis El Manar, 2092 Tunis, Tunisia

**Keywords:** geothermal springs, haloarchaea, *Halogranum*, metataxonomic, oases

## Abstract

This study assesses halophilic archaea’s phylogenetic diversity in southern Tunisia’s geothermal water. In the arid southern regions, limited surface freshwater resources make geothermal waters a vital source for oases and greenhouse irrigation. Three samples, including water, sediment, and halite soil crust, were collected downstream of two geothermal springs of the Ksar Ghilane (KGH) and Zaouet Al Aness (ZAN) oases, Tunisia. The samples were subjected to 16S rRNA gene sequencing using the Illumina Miseq sequencing approach. Several haloarchaea were identified in the geothermal springs. The average taxonomic composition revealed that 20 out of 33 genera were shared between the two geothermal sources, with uneven distribution, where the *Halogranum* genus was the most represented genus with an abundance of 18.9% and 11.58% for ZAW and KGH, respectively. Several unique site-specific genera were observed: *Halonotius, Halopelagius, Natronorubrum*, and *Haloarcula* in ZAN, and *Haloprofundus, Halomarina, Halovivax, Haloplanus, Natrinema, Halobium, Natronoarchaeum*, and *Haloterrigena* in the KGH pool. Most genus members are typically found in low-salinity ecosystems. These findings suggest that haloarchaea can disperse downstream from geothermal sources and may survive temperature and chemical fluctuations in the runoff.

## Teaser

Metataxonomic analysis reveals diverse communities of halophilic archaea in two geothermal oases in the southern Tunisian Sahara, highlighting their resilience to extreme environmental conditions.

## Introduction

Geothermal springs are formed by naturally heated groundwater. It has been found that geothermal springs harbour fascinating microbial communities adapted to high-temperature conditions. This has attracted significant interest in studying taxonomy, adaptive mechanisms, and ecological roles of microbial diversity in these environments (Kanokratana et al. 2004, Kato et al. 2009, Sayeh et al. 2010, Ghilamicael et al. 2017, Schuler et al. 2017, Massello et al. 2020).

Environmental DNA analyses from geothermal springs have shown a relative abundance of *Thermoproteota* and *Nitrososphaerota* phyla (Burton and Norris 2000, Benlloch et al. 2001, Robertson et al. 2005, Huang et al. 2011, Song et al. 2013). Interestingly, some studies have also revealed that geothermal springs contain halophilic archaea (members of the class *Halobacteria*), which can tolerate low salinity and high temperature (Elshahed et al. 2004a,b, Savage et al. 2007, Savage et al. 2008, Ghilamicael et al. 2017). Some haloarchaea were recovered from hypersaline ecosystems, such as salt deposits and solar salterns, and are known to withstand a wide range of salinity (0.3%–30% NaCl) (Kim et al. 2011, Najjari et al. 2015, 2021). Moreover, haloarchaea members were identified in low-salinity environments as brackish waters, sediments, and forest soils (Elshahed et al. 2004a, Rodriguez-Valera et al. 1979, Munson et al. 1997, Purdy et al. 2004, Savage et al. 2007, 2008, Youssef et al. 2012, Najjari et al. 2015).

Haloarchaea have adapted to survive in saline environments by two mechanisms: (i) uptake and accumulation of high concentrations of inorganic ions such as K^+^ (‘salt-in strategy’) or (ii) by synthesis and/or uptake of highly soluble organic solutes that do not interfere with intracellular enzymatic activities and cellular processes (‘salt-out strategy’) (Roberts 2005, Becker et al. 2014, Youssef et al. 2014, Najjari et al. 2015). Furthermore, haloarchaea can withstand high temperatures in natural environments, and some have an optimal growth temperature above 50°C, while others only retain their enzymatic activity at high temperatures. Examples include *Natrinema thermophila* (66 °C), *Natrinema pellirubrum* (57°C), *Halogeometricum borinquense* (57°C), *Natronolimnobius aegyptiacus* (55°C), and *Natronomonas pharaonis* (56°C) (Robinson et al. 2005, Munawar and Engel 2013, Kim et al. 2018).

Multiple geothermal springs are found in the southern regions of Tunisia. Due to limited surface freshwater resources and the *arid* climate in southern regions, geothermal waters are commonly used for oases and greenhouse irrigation (Ben Mohamed 2003). Yet, the salinity of geothermal water, ranging between 2 and 4.5 g/l, can damage irrigated agricultural areas by accumulating sodium salts in soils (Ben Hassine et al. 1996, Hachicha and Ben Aissa 2014). The microbial diversity of these geothermal sources was only investigated by Sayeh et al. (2010) based on 16S rRNA gene sequencing and cloning libraries, identifying some archaeal clones, and therefore, there is still very little knowledge of archaeal communities.

This study aims to evaluate the composition and diversity of the haloarchaea community in water, halitic soil samples, and sediments collected from two thermal springs in the southern Tunisian Sahara. An arid desert climate characterizes the area. The oases of Ksar Ghilane (KGH) and Zaouet Al Aness (ZAN) were the study areas. These oases contribute significantly to the conservation of biological diversity and the balance of ecosystems since they are habitats for various species of animals, plants, and other organisms adapted to arid conditions. The metataxonomic analysis was based on the high-throughput sequencing technology of the Illumina MiSeq platform, targeting specific sequences of the 16S rRNA gene.

## Material and methods

### Sampling and site descriptions

**Figure 1.**
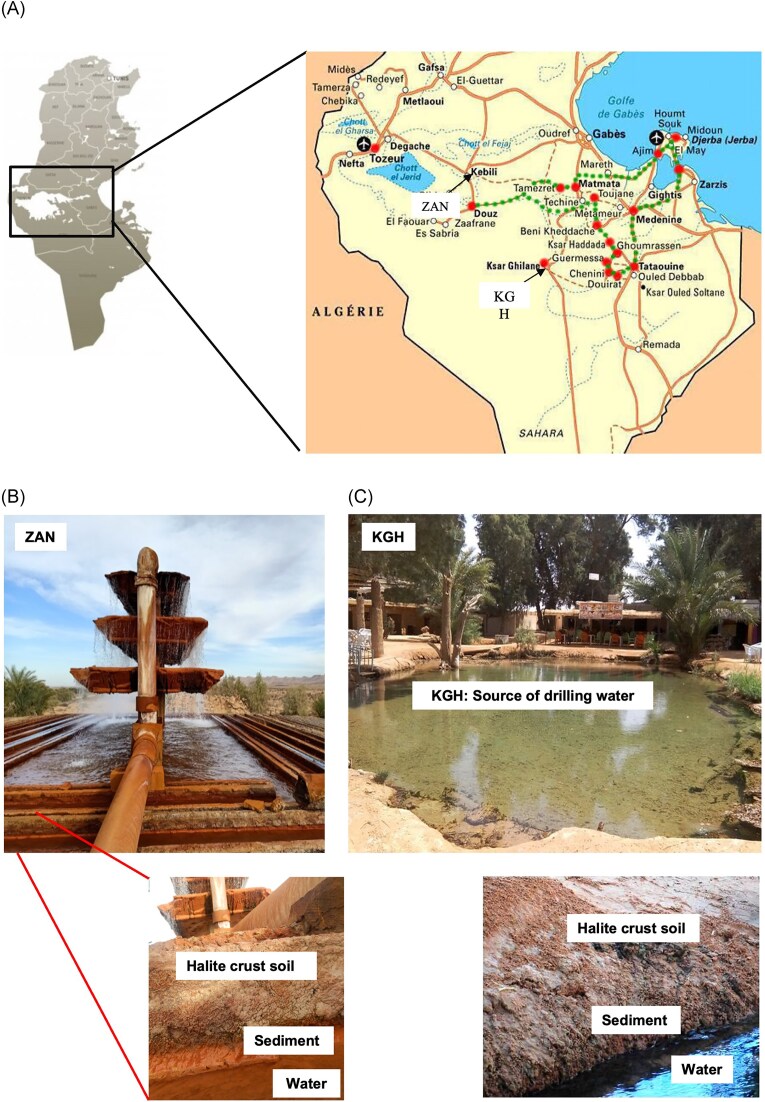
(A) Geothermal hot springs sources localization in the Tunisian map. (B) The geothermal source in Zawiet Al Aness (ZAN). (C) A pool of geothermal water in KGH.

A total of six samples (each in triplicate) were collected in December 2019 from two geothermal hot spring sources in the Kebili governorate, southwestern Tunisia (Fig. 1). Salinities were measured using a handheld SW series VistaVision refractometer (VWR, Radnor, PA, USA). Temperature and pH were recorded on site, and ∼100 g (of sediment or halite crust soil) and 1000 ml (of water) were sampled into sterile containers, placed on ice, and transported to the laboratory, where the samples were kept frozen (−20°C) until DNA extraction.

The first source is the deep geothermal drill of Zaouiet El Anes (ZAN) (33°47′30.69″N; 8°47′22.03″E; 2358 m deep) in the Souk Lahad delegation (Fig. 1A and B). The pH of drilling waters was neutral (∼7), the salt content was 4.5 g/l, and the temperature ranged from 45°C downstream to 71°C at the top of the geothermal source. Water sources are used for irrigation of oases and bathing purposes. Samples identified as S35 (sediment, 10 cm from the surface), S33 (water, 50 cm from the bottom), and S36 (halite crust soil) were taken from downstream of the geothermal source where the temperature of the water is about 45°C.

The second source is the geothermal drill site of KGH oasis (32°59′18.04″N; 9°38′22.25″E; 580 m deep) located in the middle of dunes of Douz delegation (Fig. 1A and C). As with the first site, water sources of KGH are used for irrigation of oases and bathing purposes. The water of the source was characterized by a temperature of 42°C, pH 7.5, and salt content of 4.5 g/l. From this source, three samples were taken from the edge of the KGH water pool (Fig. 1A and C) and were identified as S20 (sediment, 20 cm from the surface), S23 (50 cm from surface water), and S24 (halite crust soil).

### Physicochemical parameters of water and sediment samples

The physical parameters, including temperature, pH, water salinity, and electrical conductivity, were measured *in situ* using handheld analysing kits (digital conductivity meter 3510 WTW, pH meter). Calcium (Ca^2+^) and magnesium (Mg^2+^) concentrations were determined using direct atomic absorption spectrophotometry (PerkinElmer). Sodium (Na^+^) and potassium (K^+^) levels were determined by flame spectrophotometry using a Fisher Scientific instrument, with ion standards ranging from 1 to 100 mg/l. Chloride (Cl^−^) concentrations were determined by silver nitrate titration, employing potassium chromate or eosin/fluorescein solution as indicators. The bicarbonate (HCO_3_^−^) content was measured using a Thermo Scientific Orion 9502BNWP Carbon Dioxide Electrode according to the manufacturer’s instructions. Sulphate (SO_4_^2−^) levels were assessed using a turbidimetric method (Wimberley 1968).

### DNA extraction, PCR amplification, sequencing, and analysis

DNA was extracted from sediment and halite crust soil samples (500 mg) and filtered water samples (200 ml) using a PowerSoil DNA extraction kit (MoBio, Carlsbad, CA, USA), according to the manufacturer’s instructions. The extracted DNA was then quantified with a Qubit fluorometer (Life Technologies, Grand Island, NY, USA).

Halobacteria community structure was targeted using Halobacteria-specific primers 287F (5′-AGG TAG ACG GTG GGG TAA C-3′) and 589R (5′-RGC TAC GRA CGC TTT AGG C-3′) (Najjari et al. 2015). PCR analysis was performed in 50-µl reaction mixtures that contained 100 ng of the extracted DNA, ×1 PCR buffer (Fermentas), 2.5 mM MgSO_4_, a 0.2 mM deoxyribonucleoside triphosphate (dNTP) mixture, 0.5 U of Taq DNA polymerase (Fermentas), and a 10 µM concentration of each of the forward and reverse primers. PCR was carried out according to the following protocol: initial denaturation at 95°C for 5 min, followed by 35 cycles of denaturation at 95°C for 45 s, annealing at 55°C for 1 min, and elongation at 72°C for 1 min. A final elongation step at 72°C for 10 min was included.

Sequencing libraries were prepared using the Nextera XT Kit according to the Illumina Supporting Guide (Klindworth et al. 2012). Libraries were then multiplexed and sequenced on the MiSeq paired-end Illumina platform (Illumina, San Diego, CA, USA).

### Bioinformatics analysis for taxonomic classification

The Mothur software (v.1.30.2) was used for reads analysis (Schloss et al. 2009). Sequences were classified into operational taxonomic units (OTUs at 0.03 cutoff) using the SILVA non-redundant v138 training set (Quast et al. 2012). Rarefaction curves were computed. The number of OTUs at the genus level was assessed to describe alpha diversity between the groups. Alpha diversity indices corresponding to variables Good’s coverage, Shannon, Chao1, and Simpson were conducted using Mothur software. The Shannon and Simpson α-diversity indices were applied to estimate the diversity for each group using the Wilcoxon rank-sum test (Yoon et al. 2017). Beta diversity was calculated with Bray–Curtis distances based on the taxonomic abundance profiles. Permutational multivariate analysis of variance (PERMANOVA) was applied to measure the statistical significance of β-diversity (Yoon et al. 2017). The significance level for all statistical tests was 5% (*P* < 0.05). Principal component analysis (PCA) was carried out on the estimated archaea abundance and the chemical/physical properties of the three sample types taken from ZAN and KGH using Python 3.8.

## Results and discussion

This study reveals new insights into the diversity of haloarchaeal communities in the geothermal oases of KGH and ZAN in southern Tunisia. The differences in their physicochemical parameters, such as temperature, pH, and chemical composition, affect the haolarchaeal diversity. Although some genera were found in both cases, the dominance of *Halogranum* is notable, indicating its high adaptability to different environmental conditions.

### Physicochemical parameters of water and sediment samples

Salinity, temperature, pH, conductivity, dry residue, and the concentrations of the ions Ca^2+^, Mg^2+^, Na^+^, K^+^, SO_4_^2−^, Cl^−^, NO_3_^−^, and HCO_3_^−^ were determined from the oases samples, KGH and ZAN (Table 1). The salinity of water, sediment, and halite crust samples was within the expected range for oases, with KGH showing slightly higher values than ZAN. The temperature values were also within the range expected for arid environments, with KGH having a higher temperature (43.2°C) than ZAN (38°C). The pH values were slightly alkaline for both oases, with ZAN having a slightly higher pH (7.8) than KGH (pH = 7.6). The conductivity values are higher for ZAN (compared to KGH), although KGH has higher concentrations of the ions analysed. The concentrations of Ca^2+^, Mg^2+^, Na^+^, Cl^−^, and SO_4_^2−^ were higher in KGH than in ZAN, while K^+^ concentration was higher in ZAN. Both oases had low concentrations of NO_3_^−^. The dry residue values for both water and sediment samples were comparable for both oases, indicating a similar level of dissolved solids. Overall, the physicochemical characteristics of the two oases show some differences in the ion composition of their water and sediment samples. These differences may result from geological, hydrological, and biological variations between the two oases. Variations in the physicochemical properties of the water from the 10 other Tunisian geothermal springs located in the north and south of Tunisia have been reported, the majority of which are neutral (pH = 6.2–7.73), and two are slightly alkaline (pH = 8–8.44). Salinity varies from 3 to 18 g/l NaCl. Temperatures range from 37.5 to 73°C. In general, the springs with the highest temperatures had the lowest salinity. The composition of ions such as Ca^2+^, Mg^2+^, Na^+^, K^+^, SO_4_^2−^, Cl^−^, NO_3_^−^, and HCO_3_^−^ in the low-salinity geothermal springs of southern Tunisia is similar to that of the KGH and ZAN springs (Sayeh et al. 2010).

**Table 1.** Physical and chemical composition of samples taken at KGH and ZAN, together with salinity and temperature.

	KGH			ZAN		
Parameter	Water	Sediment	Halite crust	Water	Sediment	Halite crust
Salinity (g/l)	5.7	4.7	20.02	4	4.2	18.03
Temperature (°C)	43.2	43.2	43.2	38	38	38
pH	7.6	7.6	7.7	7.8	7.8	7.8
Conductivity (ms/cm)	5.7	6	4.75	6.74	6.13	3.23
Dry residue (mg/l)	3853	3770	28 547	3853	3750	3895
Ca^2+^ (mg/l)	483	320	480	337.6	320	458
Mg^2+^ (mg/l)	237.6	226.8	302.5	171.9	222	287.05
Na^+^ (mg/l)	849.3	850.03	1302	386.4	333.5	1242
K^+^ (mg/l)	19	20.1	45	51.48	46.02	38.9
SO_4_^2−^ (mg/l)	1561	1102.02	500.3	996	859,2	400.85
Cl^−^ (mg/l)	1517.7	1429.5	1800.2	709	1057.9	1789.3
NO_3_^−^ (mg/l)	0.1	No detected	No detected	0.4	6.1	No detected
HCO_3_^−^ (mg/l)	384	393.7	520	115	384.4	485.2

### Haloarchaeal community structure

A significant variation in haloarchaeal diversity was observed between the geothermal springs of KGH and ZAN, with higher species richness at KGH. Unique genera were identified at each site, indicating possible ecological adaptations to the distinct physicochemical conditions.

**Figure 2.**
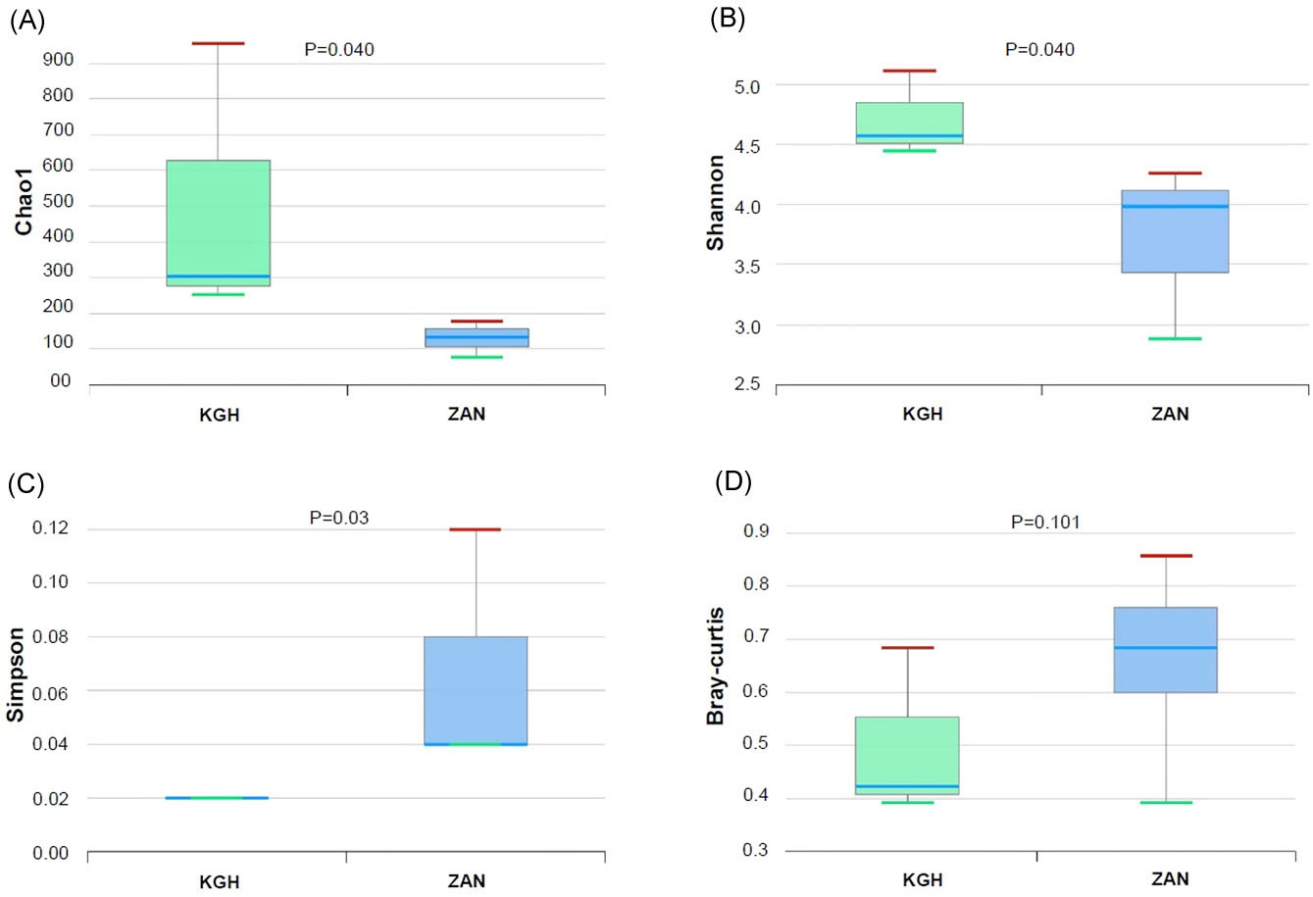
Comparison of species richness and α-, β-diversities in haloarchaeal taxonomic profile between ZAN and KGH samples sets by (A) Chao1, (B) Shannon, (C) Simpson, and (D) Bray–Curtis indices. The Shannon and Simpson α-diversity indices were applied to estimate the diversity for each group using the Wilcoxon rank-sum test. Beta diversity was calculated with Bray–Curtis distances based on the taxonomic abundance profiles. Permutational multivariate analysis of variance (PERMANOVA) was applied to measure the statistical significance of β-diversity.

A total of 29 067 valid reads of 16S rRNA V3-V4 region amplicon produced by Illumina sequencing were clustered in 1853 OTUs. The variation in the haloarchaeal community across samples was assessed by analysing diversity indices, including Chao1, Shannon, Simpson, and Good’s library coverage (Table 2). The sequence data represented a diverse haloarchaeal community of 622 and 371 OTUs for KGH and ZAN samples, respectively (3% divergence). Rarefaction curves and good coverage with values >98% for all samples indicated excellent OTU coverage afforded by the deep sequencing (Supplementary Fig. S1). Species richness and diversity index values among KGH and ZAN sets were compared and statistically tested (Fig. 2). Results showed that the species richness of KGH (252–954.17) samples was higher than ZAN (77.59–177.66) as measured by Chao 1 (*P* = 0.04) (Table 2). Similar patterns were observed in the diversity index, calculated using the Shannon index (5.12 for KGH vs. 4.26 for ZAN) and the Simpson index (0.2 for KGH vs. 0.4 for ZAN), which showed significant differences between the two sites at the genus level (Fig. 2). These differences may be due to the chemical composition of the waters since the water from the KGH site is more mineralized than that from the ZAN site (Table 1).

**Table 2.** Alpha-diversity of the haloarchaeal communities associated with KGH and ZAN samples.

Sample name	Target reads	OTUs	Chao1	Shannon	Simpson	Good’s coverage of library (%)
S23_ground water_KGH	15 325	953	954.17	5.12	0.02	99.82
S20_sediment_KGH	6 518	296	303.56	4.57	0.02	99.57
S24_halite crust soil_KGH	2 909	233	252.02	4.45	0.02	98.62
S33_ground water_ZAN	1 694	127	135.05	3.98	0.04	98.94
S35_sediment_ZAN	1 242	73	77.59	2.88	0.12	98.95
S36_halite crust soil_ZAN	1 379	171	177.66	4.26	0.04	98.33

### Taxonomic profiling of haloarchaea

**Figure 3.**
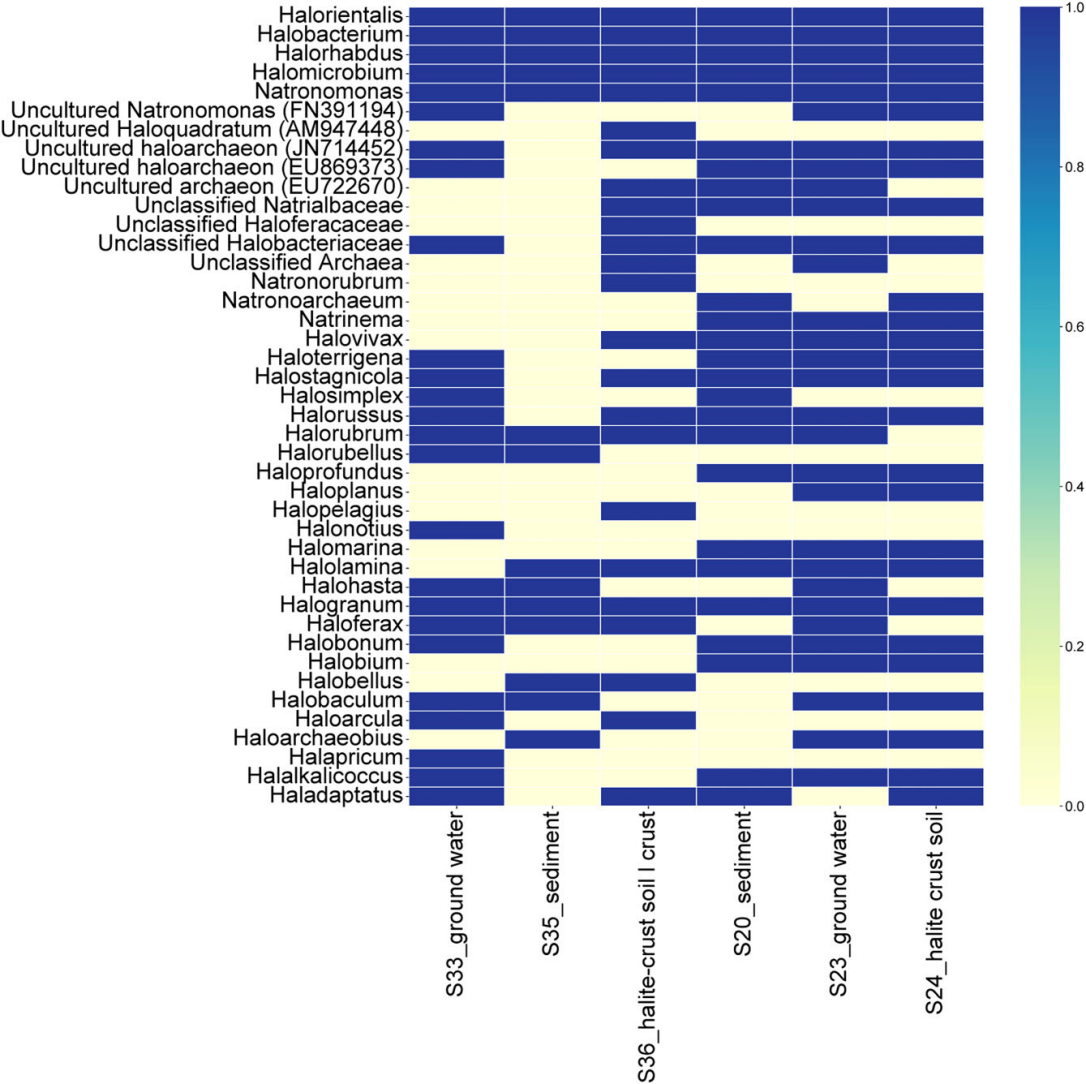
The heat map shows the genera’s presence/absence distribution across all samples from KGH and ZAN sites.

The genera distribution found in samples from geothermal spring sources (ZAN and KGH), assessed based on Halobacteria-specific gene amplifications, are shown in Fig. 3 and Supplementary Table S1.

### ZAN samples

**Figure 4.**
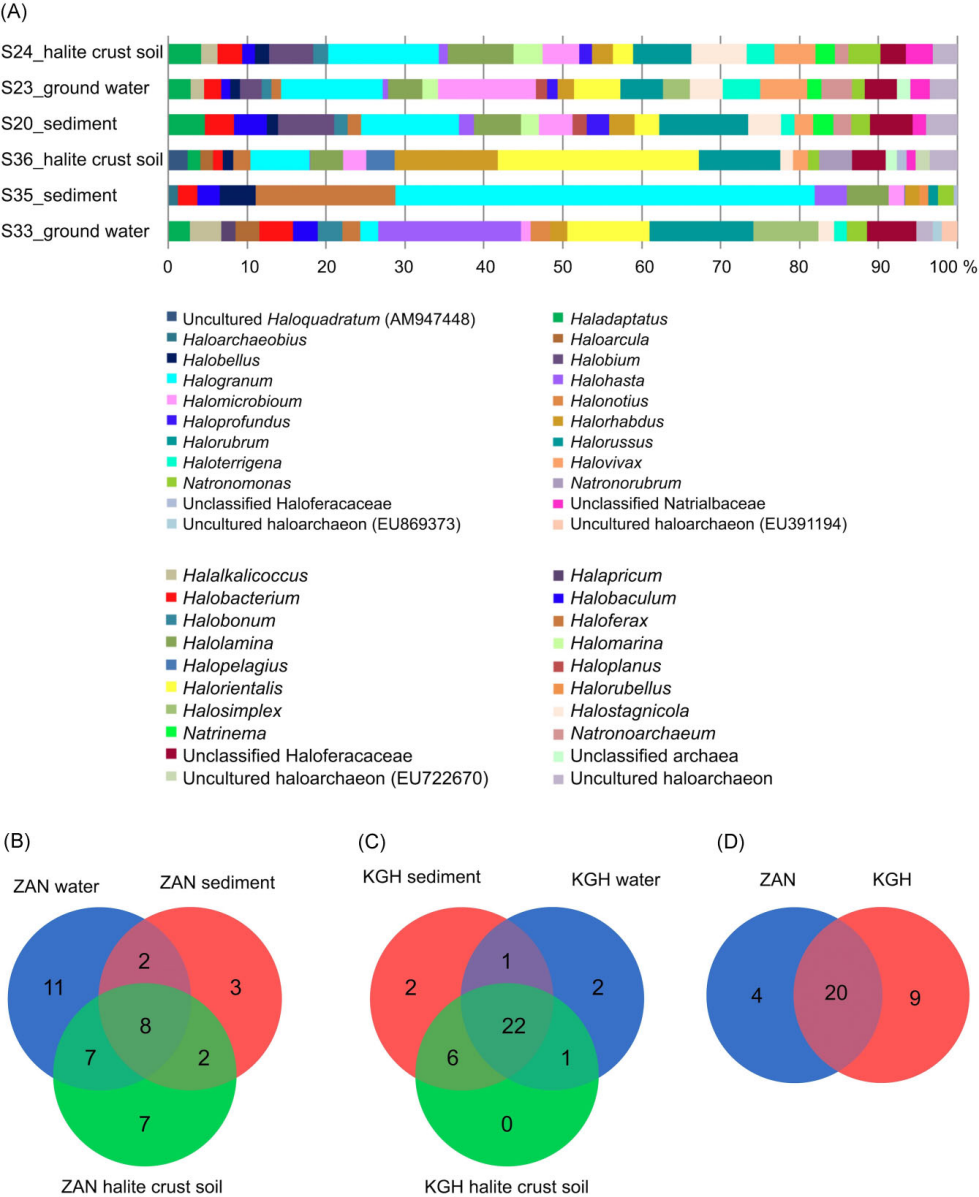
Taxonomic abundances. (A) Bar plots showing the genus taxonomic abundance distributed across the samples. Taxa with low abundance (<1%) were eliminated. Diagram representing the distribution of standard and unique OTUs (3% distance level) across samples of (B) ZAN source, (C) KGH samples, and (D) across average taxonomic distribution within KGH and ZAN geothermal spring sources.

A total of 4315 valid reads amplicons was produced with an average length of 280 bp across 3 samples ranging from 150 to 300 bp (Table 2). Taxonomic level distribution showed that OTUs of all samples (*n* = 3) were affiliated to the *Methanobacteriota* phylum and *Halobacteria* class and the order *Halobacteriales*. At the genus level, a total of 33 genera were identified out of 76 halophilic archaea genera presently described (Parte et al. 2020, Cui et al. 2023) and distributed as follows:

- In the groundwater sample (S33), a total of 22 genera have been identified with a net predominance of *Halohasta* (16.64%) (Fig. 4A, Supplementary Table S2). The remaining genera members were <10% in relative abundance. The genus *Halohasta*, a haloalkaliphilic archaeon, was first identified in 2012 and includes three species: *Halohasta litorea*, isolated from a brine sample from an aquaculture farm in Daliang, China; *Halohasta litchfieldiae*, isolated from a surface water sample from Deep Lake, Antarctica (Mou et al. 2012); and *Halohasta salina*, isolated from inland saline soil, Xinjiang, China (Cheng et al. 2023).
- Within the halite soil crust sample (S36), 17 genera were identified with the occurrence of *Halorientalis* genus members (22.19%) (Fig. 4A, Supplementary Table S2). The remaining genera comprise <6% of the relative abundances. The genus *Halorientalis* was proposed by Cui et al. (2011), including seven species: *Halorientalis brevis, Halorientalis hydrocarbonoclasticus, Halorientalis pallida, Halorientalis persicus, Halorientalis regularis, Halorientalis litorea*, and *Halorientalis marina* (Parte et al. 2020). These species have been isolated from saline environments and from freshwater from the Liaohe estuary (China) (Durán-Viseras et al. 2019, Shi et al. 2021, Wang et al. 2022).
- In the sediment sample (S35), 14 genera were identified, with the occurrence of sequences affiliated with *Halogranum* genus members being the most abundant (48.23%), followed by *Haloferax* (16.02%) (Fig. 4A, Supplementary Table S2). The relative abundance of remaining genera did not reach 5% of classified reads. The occurrence of the *Halogranum* genus in low-salinity habitats was previously reported (Youssef et al. 2012, Najjari et al. 2015). *Halogranum* species include *Halogranum amylolyticum, Halogranum gelatinilyticum* (Cui et al. 2011), and *Halogranum salarium* (Kim et al. 2011), which is recently re-classified as a heterotypic synonym of *Halogranum rubrum* (Cui et al. 2010, Tan et al. 2024). The two species *Hgn. rubrum* and *Hgn. gelatinilyticum* have been previously isolated from marine solar salterns and evaporitic salt crystals collected along the seashore of Namhae, South Korea (Cui et al. 2010, 2011, Kim et al. 2011).

The nature and prevalent salinities in these samples suggest the capability of the genus *Halogranum* to survive in environments of varying salinities (Youssef et al. 2014, Najjari et al. 2015, Shi et al. 2021). It is noteworthy that eight main genera were common to all samples, comprising *Halobacterium, Halorhabdus, Halomicrobium, Haloferax, Halorientalis, Halorubrum, Halogranum*, and *Natronomonas*, the majority of which are widespread, ranging from salt-saturated habitats to low-saline environments, as well as from hot spring sources (Elshahed et al. 2004a,b, Purdy et al. 2004, Sayeh et al. 2010, Youssef et al. 2014, Najjari et al. 2015, 2021).

A further comparison of the haloarchaea genera members indicated that three samples (water, halite crust soil, and sediment) shared 8 common genera, including *Halobacterium, Halorhabus, Halomicrobium, Haloferax, Halorientalis, Halorubrum, Halogranum*, and *Natronomonas* (Fig. 4B, Supplementary Table S1). Genera unique to individual samples were also observed, including *Halalkalicoccus, Halobonum, Halonotius, Halosiccatus, Halapricum, Halomicroarcula, Haloterrigena, Halosimplex*, and uncultured haloarchaeaon (FN391194, EU869373, JN714428), which were only detected in the water sample. In contrast, *Halopelagius, Natronorubrum*, and *Halovivax* (plus some unclassified and uncultured genera) were detected only in the halite soil crust (Fig. 4B, Supplementary Table S1). Finally, *Haloarchaeobius, Halorubellus*, and an uncultured haloarchaeon (EF533953) were specific to the sediment samples (Fig. 4C, Supplementary Table S1).

### KGH source

A total of 24 752 valid reads amplicons was produced with an average length of 282 bp across three samples ranging from 100 to 300 bp (Table 2). Similar to the ZAN pool, the OTUs of all datasets were affiliated with the *Methanobacteriota* phylum and *Halobacteria* class Order *Halobacteriales*.

At the genus level, 27 genera were identified in all samples with unequal distribution (Fig. 4). Datasets of three samples contained one primary genus member, the *Halogranum*, with a relative abundance of ∼12%. Indeed, a total of 18 genera (*Halomarina, Halostagnicola, Halolamina, Halalkalicoccus, Halobacterium, Halobonum, Halorhabdus, Halomicrobium, Halorientalis, Haladaptatus, Halorussus, Halovivax, Halorubrum, Natrinema, Halogranum, Halobium, Natronomonas, Haloterrigena*) and unclassified *Halobacteriaceae*, unclassified *Natrialbaceae*, and uncultured haloarchaeon (JN714452; EU869373) were common in all KGH samples (Fig. 4B, Supplementary Table S1).

### Average taxonomic composition of two geothermal sources

**Figure 5.**
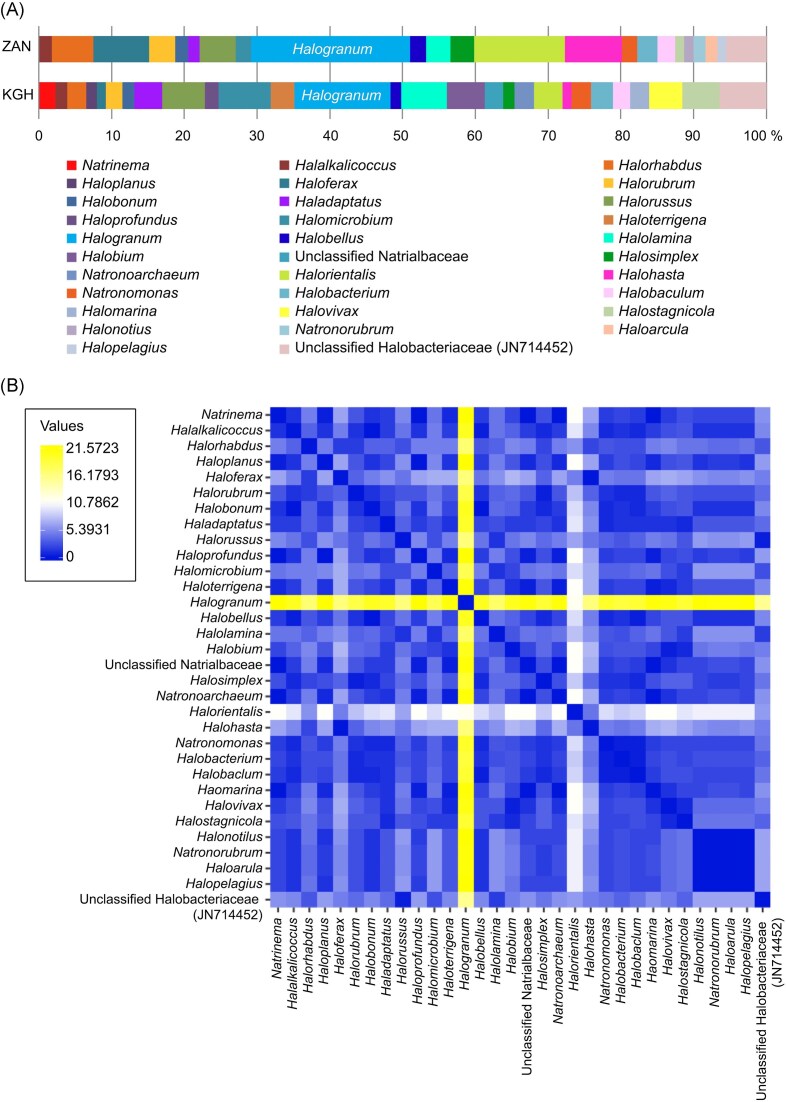
Average taxonomic composition. (A) Bar plots show genus members' average taxonomic composition across the ZAN and KGH sites. Taxa with low abundance (<1%) were eliminated. (B) A hierarchical clustering dendrogram based on a distance matrix of average taxonomic composition at the genus level in KGH and ZAN sites shows the abundance of *Halogranum* genus members compared to other genera.

The average taxonomic compositions of shared and unique archaeal taxa between the two datasets were assessed (Fig. 5). Results showed noticeable differences in haloarchaeal communities’ distribution between the two geothermal sites.

Among the 33 detected genera, 20 were encountered in the two geothermal sources investigated, with uneven distribution (Fig. 5A). It is worth noting that *Halogarnum* sequences were the most represented across the two sites with an abundance of 18.9% and 11.58% for ZAN and KGH sites, respectively (Fig. 5B). The remaining genera, including *Halostagnicola, Halolamina, Halalkalicoccus, Halobacterium, Halobonum, Halorubrum, Halohasta, Halobaculum, Halorhabdus, Halomicrobium, Haloferax, Halorientalis, Natronomonas, Haladaptatus, Halorussus, Halosimplex, Halobellus*, unclassified *Halobacteriaceae*, and uncultured haloarchaeon (JN714452), represented <10% of the haloarchaeal community. The genera *Halonotius, Halopelagius, Natronorubrum*, and *Haloarcula* were detected only in ZAN samples. In contrast, eight genera were detected only in KGH samples, comprising *Haloprofundus, Halomarina, Halovivax, Haloplanus, Natrinema, Halobium, Natronoarchaeum, Haloterrigena*, and several unclassified *Natrialbaceae* members. Almost all the above genera were previously identified in low-salinity environments. In a survey of a low-salt, sulphide, and sulphur-rich spring in southwestern Oklahoma, Elshahed, and collaborators isolated halophilic archaeal clones assigned to *Halogeometricum, Natronomonas, Halococcus*, and *Haloferax* genus members (Elshahed et al. 2004a). The salinity of brine and sediment samples from which these archaea were isolated ranged between 0.7% and 1%. Similarly, *Haloferax* and *Halogeometricum* members have been isolated from low-salinity estuary sediments (southeast of Great Britain) where pore-water salinity was close to that of seawater (∼2.5% NaCl) (Purdy et al. 2004). *Haloarcula* and *Halobacterium* members have been isolated from low-salinity coastal sediments and brine of Goa (Braganca and Furtado 2009). Recently, Kimbrel et al. (2018) have illustrated the identification of halophilic archaea in brine and sediment (7.5% NaCl) collected from a solar salt pond (San Francisco, CA, USA). Some haloarchaea clones were also detected in another Tunisian study, which used 16S rRNA gene libraries to evaluate a Mahassen geothermal source in southern Tunisia (Sayeh et al. 2010). Notably, Mahassen, ZAN, and KGH springs form part of the Continental Intercalary (CI) aquifer of south Algeria and Tunisia (Abid et al. 2009).

The results suggest that members of *Halogranum* sequences are the most abundant genus collected from KGH and ZAN geothermal sources. To our knowledge, no studies describe the survival mechanisms that enable such microorganisms to thrive simultaneously under low salinity and high-temperature conditions. Possibly, the existence of haloarchaea members may be the result of water diffusing to the borders of the hot spring water channel, where its evaporation may induce a reduction in humidity and an increase in salinity in the top layers of the soil, thus providing a suitable environment for halophilic archaea. Further work is recommended to test such a hypothesis.

To thrive in a low-salt environment, halophilic archaea use a variety of organic solutes to counter external osmotic pressure, including amino acids and derivatives, polyols and derivatives, sugars (trehalose), betaines, and ectoine (Youssef et al. 2014, Najjari et al. 2015, 2023). Indeed, a correlation between intracellular accumulation of compatible solutes and growth at supra-optimal temperatures has been observed in halotolerant or slightly halophilic (hyper) thermophiles, indicating that these solutes could act in a thermostabilizing function (Da Costa et al. 1998; Hensel and König 1988, Martins and Santos 1995, Ramos et al. 1997, Pais et al. 2005).

**Figure 6.**
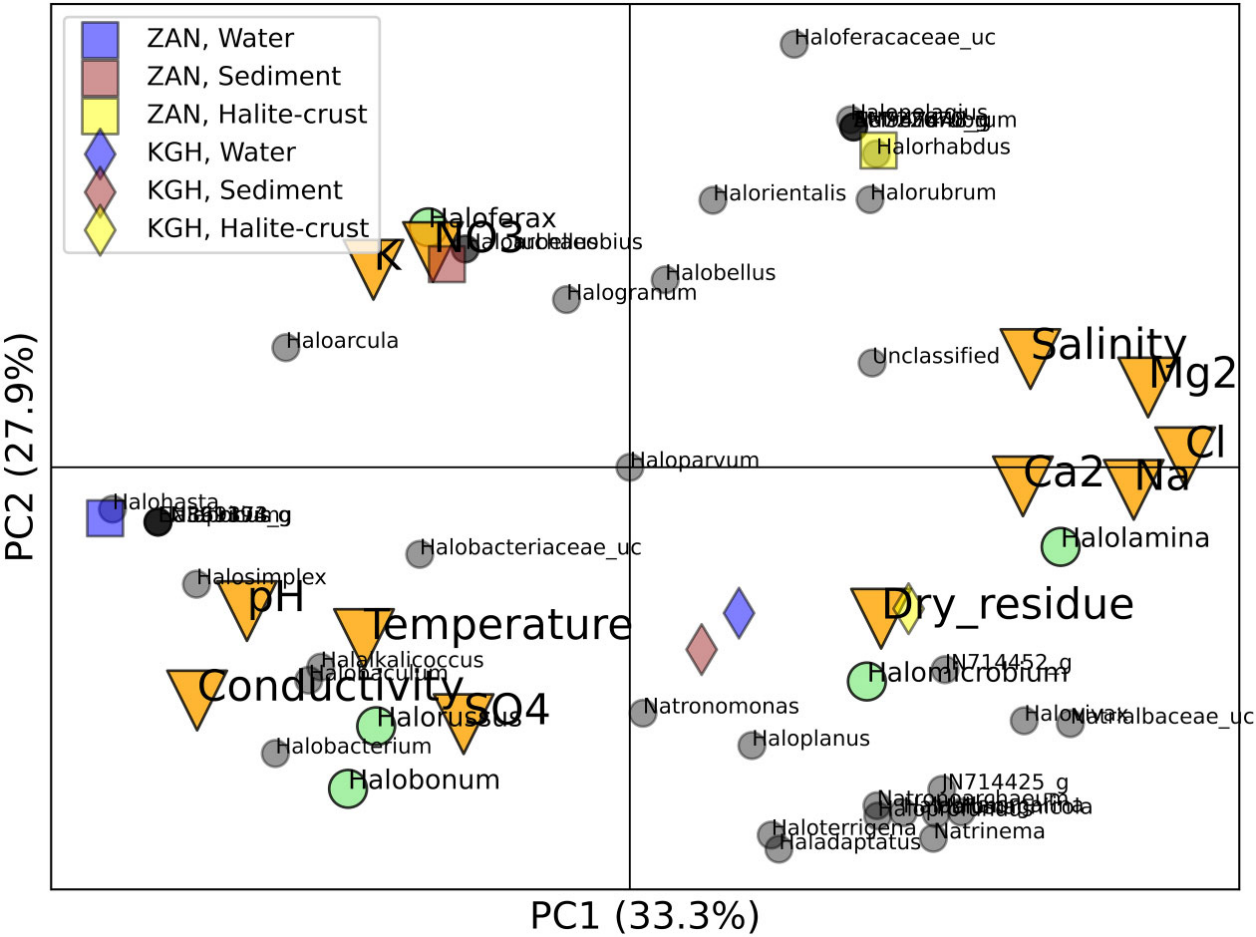
PCA analysis of the chemical/physical measures (triangles) and Archaea content (circles) in oases ZAN (squares) and KGH (diamonds) at three sample types—water, sediment, and halite crust. PC1 explains the majority of variance in the dataset along the x-axis, while PC2 has the second most variance along the y-axis. Created using Python 3.8 and Matplotlib.

The PCA can show non-statistical co-associations between the Archaea taxa, the chemical/physical markers, the oases, and sample types (Fig. 6). Strong variation was seen between the ZAN samples in terms of both chemical/physical measures and taxa. The ZAN halite crust samples were high in such measures as salinity, Na^+^, Cl^−^, Mg^2+^, and Ca^2+^ and had a relatively high abundance of the extremely halophilic archaeal*, Halolamina*. In contrast, the ZAN water samples were higher in pH, conductivity, SO_4_^2−^, and temperature and had a relatively high abundance of *Halorussus* and *Halobonum*. ZAN sediment samples had the general characteristics of both the halite crust and water samples, were also relatively high in K^+^ and NO_3_^−^, and had a relatively high occurrence of *Haloferax*.

The KGH samples were much more similar to each other and, in general, were most associated with dry residue and taxa such as *Halomicrobium*. PC1, which accounted for 33.3% of the variance, appeared to be generally explained by salinity-alkalinity differences, while PC2 accounted for 27.9% of the total, but it is less clear what factors influence this dimension.

Few researchers are dedicated to deciphering the haloarchaeal community in high-temperature environments with low salinity concentration, both with culture-dependent and independent methods. Here, this is the first report that reveals the existence of haloarchaeal-related members with the occurrence of the genus *Halogranum* in two geothermal sources. This genus is rarely documented and has been reported more in low-salinity environments (Youssef et al. 2012, Najjari et al. 2015). The prevalence and distribution of *Halogranum* suggest possessing a yet-unknown mechanism for resisting lower salt and high temperature. These results indicate that the ecological range of these physiologically versatile microorganisms is much broader than previously believed.

## Conclusions

Haloarchaea diversity in halite crusted soil, groundwater, and sediments from the KGH and ZAN geothermal oases in the southern Tunisian Sahara showed different taxonomic profiles. The genus *Halogranum* dominates in both oases. On the other hand, *Haloprofundus, Halomarina*, and *Haloterrigena* were found only in KGH, while *Halonotus, Halopelagius*, and *Natronorubrum* only in ZAN, suggesting ecological adaptations to the different physicochemical characteristics of the oases. Further studies using whole-genome sequencing could reveal insights into the mechanisms that enable haloarchaea to colonize saline geothermal springs.

## Supplementary Material

fnae106_Supplemental_File

## Data Availability

The obtained raw sequences were submitted to the National Center for Biotechnology Information (NCBI) as Sequence Read Archive (SRA) under accession numbers as follows: S33 (SRR23374283), S36 (SRR23423409), S35 (SRR23423408), S23 (SRR23423493), S20 (SRR23423410), and S24 (SRR23423411).
