# Supplementary material for: Metataxonomic analysis of halophilic archaea community in two geothermal oases in the southern Tunisian Sahara
Source: FEMS Microbiol Lett. 2024 Dec 5;372:fnae106. doi: 10.1093/femsle/fnae106 (PMC11719619; doi:10.1093/femsle/fnae106)
Supplement: fnae106_Supplemental_File [file fnae106_supplemental_file.docx]

**Metataxonomic analysis of halophilic archaea community in two geothermal oases in the southern Tunisian Sahara**

Afef Najjari^1^, Khaled Elmnasri^2^, Hanene Cherif^2^, Stephen Burleigh^3^, Amel Guesmi^2^, Mouna Mahjoubi^2^, Javier A. Linares-Pastén^4*^, Ameur Cherif^2^, Hadda-Imene Ouzari^1^

^1^Faculté des Sciences de Tunis, LR03ES03 Laboratoire de Microbiologie et Biomolécules Actives, Université Tunis El Manar, 2092 Tunis, Tunisia;

^2^Higher Institute for Biotechnology, University Manouba, BVBGR-LR11ES31, Biotechpole Sidi Thabet, 2020 Ariana, Tunisia;

^3^ Department of Process and Life Science Engineering, Lund University, Lund, Sweden

^4^ Division of Biotechnology, Faculty of Engineering, Lunds Tekniska Högskola (LTH), Lund University, P. O. Box 124, 22100 Lund, Sweden

**Corresponding author:**

e-mail: javier.linares_pasten@biotek.lu.se


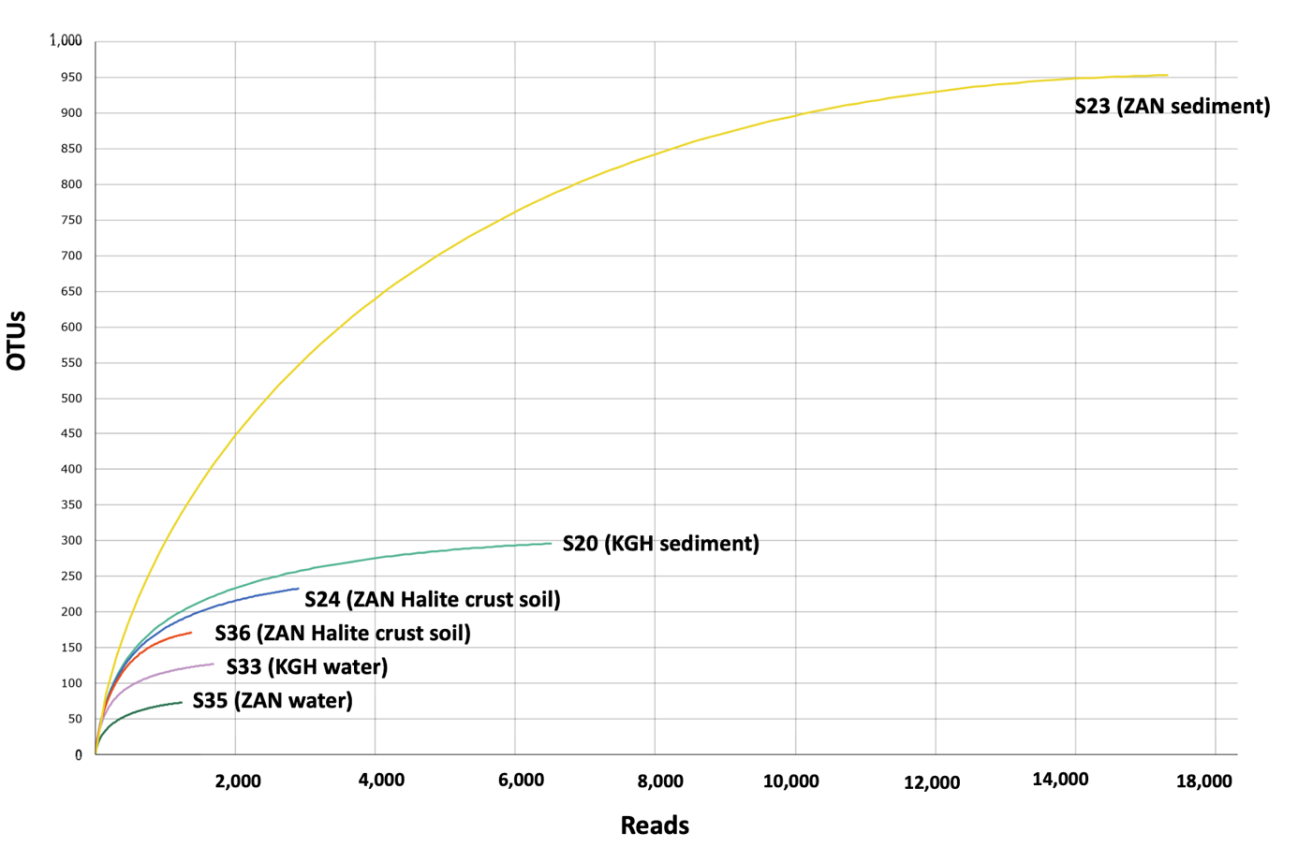


**Figure. S1** Genus-level rarefaction curves of samples based on the 16S rDNA sequences. OTUs are operation taxonomic units.

**Table S1.** Genera present or missing in all samples.

|  |  | **ZAN** | | | **KGH** | | |
| --- | --- | --- | --- | --- | --- | --- | --- |
|  | **Genus** | **Ground**  **water (S33)** | **Sediment**  **(S35)** | **Halite-crust**  **soil (S36)** | **Ground**  **water (S33)** | **Sediment**  **(S35)** | **Halite-crust soil (S36)** |
| 1 | *Halobacterium* | X | X | X | X | X | X |
| 2 | *Halorhabdus* | X | X | X | X | X | X |
| 3 | *Halomicrobium* | X | X | X | X | X | X |
| 4 | *Haloferax* | X | X | X |  | X |  |
| 5 | *Halorientalis* | X | X | X | X | X | X |
| 6 | *Halorubrum* | X | X | X | X | X | X |
| 7 | *Halogranum* | X | X | X | X | X | X |
| 8 | *Natronomonas* | X | X | X | X | X | X |
| 9 | Halohasta | X | X |  |  | X |  |
| 10 | *Halobaculum* | X | X |  |  | X | X |
| 11 | *Halostagnicola* | X |  | X | X | X | X |
| 12 | *Haladaptatus* | X |  | X | X | X | X |
| 13 | *Halorussus* | X |  | X | X | X | X |
| 14 | (JN714452) Uncultured haloarchaeon | X |  | X | X | X | X |
| 15 | *Unclassified* Halobacteriaceae | X |  | X | X | X | X |
| 16 | *Halolamina* |  | X | X | X | X | X |
| 17 | *Halalkalicoccus* | X |  |  | X | X | X |
| 18 | *Halobonum* | X |  |  | X | X | X |
| 19 | *Halosimplex* | X |  |  | X |  |  |
| 20 | (EU869373) Uncultured haloarchaeon | X |  |  | X | X | X |
| 21 | (FN391194) Uncultured  *Natronomonas* | X |  |  |  | X | X |
| 22 | (EU722670) Uncultured archaeon |  |  | X | X | X |  |
| 23 | *Halovivax* |  |  | X | X | X | X |
| 24 | *Natronorubrum* |  |  | X |  |  |  |
| 25 | *Halobellus* |  | X | X |  |  |  |
| 26 | *Halonotius* | X |  |  |  |  |  |
| 27 | *Halapricum* | X |  |  |  |  |  |
| 28 | *Halorubellus* |  | X |  |  |  |  |
| 29 | *Halopelagius* |  |  | X |  |  |  |
| 30 | (AM947448) Uncultured *Haloquadratum* |  |  | X |  |  |  |
| 31 | Unclassified *Natrialbaceae* |  |  | X | X | X | X |
| 32 | *Haloarcula* | X |  | X |  |  |  |
| 33 | Unclassified *Haloferacaceae* |  |  | X |  |  |  |
| 34 | *Natronoarchaeum* |  |  |  | X |  | X |
| 35 | *Natrinema* |  |  |  | X | X | X |
| 36 | *Halobium* |  |  |  | X | X | X |
| 37 | *Halomarina* |  |  |  | X | X | X |
| 38 | *Haloarchaeobius* |  | X |  |  | X | X |
| 39 | *Haloprofundus* |  |  |  | X | X | X |
| 40 | *Haloplanus* |  |  |  |  | X | X |
| 41 | *Haloterrigena* | X |  |  | X | X | X |
| 42 | Unclassified Archaea |  |  | X |  | X |  |

**Table S2.** Genera abundance distribution found in samples from geothermal spring sources (ZAN and KGH).

|  | **ZAN** | | | **KGH** | | |
| --- | --- | --- | --- | --- | --- | --- |
| **Genus** | **Ground**  **water (S33)** | **Sediment**  **(S35)** | **Halite-crust**  **soil (S36)** | **Ground**  **water (S33)** | **Sediment**  **(S35)** | **Halite-crust**  **soil (S36)** |
| (AM947448) Uncultured  Haloquadratum | 0.00 | 0.00 | 2.10 | 0.00 | 0.00 | 0.00 |
| (EU722670) Uncultured  archaeon | 0.00 | 0.00 | 1.52 | 0.00 | 0.00 | 0.00 |
| (EU869373) Uncultured  haloarchaeon | 1.06 | 0.00 | 0.00 | 0.00 | 0.00 | 0.00 |
| (FN391194) Uncultured  *Natronomonas* | 1.83 | 0.00 | 0.00 | 0.00 | 0.00 | 0.00 |
| *Haladaptatus* | 2.48 | 0.00 | 1.38 | 4.17 | 2.44 | 3.61 |
| *Halalkalicoccus* | 3.66 | 0.00 | 0.00 | 0.00 | 1.57 | 1.82 |
| *Halapricum* | 1.71 | 0.00 | 0.00 | 0.00 | 0.00 | 0.00 |
| *Haloarchaeobius* | 0.00 | 1.13 | 0.00 | 0.00 | 0.00 | 0.00 |
| *Haloarcula* | 2.77 | 0.00 | 1.45 | 0.00 | 0.00 | 0.00 |
| (JN714425) Unclassified  *Halobacteriaceae* | 0.00 | 0.00 | 0.15 | 1.17 | 0.48 | 0.86 |
| Unclassified *Halobacteriaceae* | 5.79 | 0.00 | 3.55 | 2.68 | 3.07 | 1.93 |
| *Halobacterium* | 3.90 | 2.17 | 1.09 | 3.28 | 1.84 | 2.72 |
| *Halobaculum* | 2.89 | 2.58 | 0.00 | 3.77 | 1.06 | 1.44 |
| *Halobellus* | 0.00 | 4.19 | 1.09 | 1.24 | 1.02 | 1.55 |
| *Halobium* | 0.00 | 0.00 | 0.00 | 6.40 | 2.43 | 4.92 |
| *Halobonum* | 2.95 | 0.00 | 0.00 | 1.55 | 1.07 | 1.65 |
| Unclassified *Haloferacaceae* | 0.00 | 0.40 | 1.09 | 0.00 | 0.00 | 0.00 |
| *Haloferax* | 2.07 | 16.02 | 1.89 | 1.53 | 1.10 | 0.00 |
| *Halogranum* | 2.13 | 48.23 | 6.53 | 11.15 | 11.29 | 12.31 |
| *Halohasta* | 16.65 | 3.70 | 0.07 | 1.73 | 0.59 | 0.93 |
| *Halolamina* | 0.00 | 4.83 | 3.63 | 5.39 | 3.80 | 7.36 |
| *Halomarina* | 0.00 | 0.00 | 0.00 | 1.98 | 1.78 | 3.23 |
| *Halomicrobium* | 1.12 | 1.85 | 2.54 | 3.82 | 10.91 | 4.02 |
| *Halonotius* | 2.30 | 0.00 | 0.00 | 0.00 | 0.00 | 0.00 |
| *Haloparvum* | 0.00 | 0.00 | 0.00 | 0.00 | 0.00 | 0.00 |
| *Halopelagius* | 0.00 | 0.08 | 3.12 | 0.00 | 0.00 | 0.00 |
| *Haloplanus* | 0.00 | 0.00 | 0.00 | 1.69 | 1.23 | 0.00 |
| *Haloprofundus* | 0.00 | 0.00 | 0.00 | 2.55 | 1.13 | 1.44 |
| *Halorhabdus* | 1.95 | 1.61 | 11.39 | 2.85 | 1.83 | 2.30 |
| *Halorientalis* | 9.68 | 0.00 | 22.19 | 2.82 | 5.19 | 2.27 |
| *Halorubellus* | 0.00 | 1.05 | 0.00 | 0.00 | 0.00 | 0.00 |
| *Halorubrum* | 2.07 | 1.05 | 6.24 | 2.27 | 1.38 | 2.30 |
| *Halorussus* | 10.04 | 0.00 | 2.68 | 7.86 | 3.31 | 4.13 |
| *Halosimplex* | 7.62 | 0.00 | 0.00 | 0.00 | 3.02 | 0.00 |
| *Halostagnicola* | 1.77 | 0.00 | 1.45 | 3.76 | 3.64 | 6.15 |
| *Haloterrigena* | 1.48 | 0.00 | 0.00 | 1.53 | 4.18 | 3.06 |
| *Halovivax* | 0.00 | 0.00 | 1.60 | 2.12 | 5.23 | 4.57 |
| Uncultured haloarchaeon | 1.89 | 0.00 | 3.05 | 3.51 | 3.02 | 2.68 |
| **(**JN714452) Unclassified  *Natrialbaceae* | 0.00 | 0.00 | 1.02 | 1.56 | 2.16 | 3.03 |
| *Natrinema* | 0.00 | 0.00 | 0.00 | 2.32 | 1.58 | 2.13 |
| *Natronoarchaeum* | 0.00 | 0.00 | 0.00 | 2.06 | 3.38 | 1.55 |
| *Natronomonas* | 2.36 | 1.85 | 1.23 | 2.12 | 1.49 | 3.54 |
| *Natronorubrum* | 0.00 | 0.00 | 3.63 | 0.00 | 0.00 | 0.00 |
| Unclassified Archaea | 0.00 | 0.00 | 1.23 | 0.00 | 1.51 | 0.00 |
